# Nutraceutical Valorization of Exhausted Olive Pomace from *Olea europaea* L. Using Advanced Extraction Techniques

**DOI:** 10.3390/plants13162310

**Published:** 2024-08-20

**Authors:** Vittorio Carlucci, Maria Ponticelli, Daniela Russo, Fabiana Labanca, Valeria Costantino, Germana Esposito, Luigi Milella

**Affiliations:** 1 Department of Science, Universitá degli Studi della Basilicata, Via dell’Ateneo Lucano 10, 85100 Potenza, Italy; vittorio.carlucci@unibas.it (V.C.); maria.ponticelli@unibas.it (M.P.); daniela.russo@unibas.it (D.R.);; 2 Department of Biochemical Pharmacology & Drug Design, Institute of Molecular Biology “Roumen Tsanev”, Bulgarian Academy of Sciences (BAS), Acad. G. Bonchev Str., bl. 21, 1113 Sofia, Bulgaria; 3 BioActiPlant s.r.l., Viale Dell’Ateneo Lucano 10, 85100 Potenza, Italy; 4 The Blue Chemistry Lab Group, Department of Pharmacy, Università degli Studi di Napoli Federico II, 80131 Napoli, Italy; valeria.costantino@unina.it

**Keywords:** *Olea europaea* L., by-products, olive pomace, green chemistry, nutraceuticals, circular economy

## Abstract

Exhausted olive pomace (EOP) represents the principal residue of olive pomace. Several studies have optimized the extraction of specialized metabolites from the EOP of *Olea europaea* L., but a comparison between different extractive methods has not been made. For this reason, the present investigation aims to compare four different extractive methods by using water and 15% ethanol/water as extractive solvents. Specifically, based on extract antioxidant activity, the methods compared were maceration (MAC), microwave-assisted extraction (MAE), ultrasound-assisted extraction (UAE), and Accelerated Solvent Extraction (ASE). Between these, the UAE and ASE hydroalcoholic EOP extracts were demonstrated to have the highest antioxidant activity. Subsequently, these extracts were investigated for their hypoglycemic and antiradical activity using in vitro cell-free and cell-based assays, respectively. ASE hydroalcoholic EOP extract demonstrated the greatest ability to inhibit the α-amylase enzyme and an in vitro antioxidant activity comparable to N-acetyl cysteine in HepG2 cells. UAE and ASE extracts’ phytochemical characterization was also performed, identifying seven phenolic compounds, including 3-hydroxytyrosol, tyrosol, and, for the first time, salidroside. The ASE hydroalcoholic EOP extract was the richest from a phytochemical point of view, thus confirming its major biological activity. Therefore, ASE and 15% ethanol/water may represent the best extractive method for EOP nutraceutical valorization.

## 1. Introduction

In Europe, olive oil production represents a significant agro-industrial sector in production and consumption. The Italian olivicultural sector is among the most important in the world: its production, in fact, accounts for 15–18% of global production (second after Spain). Specifically, Italian olive tree (*Olea europaea* L.) cultivation spans approximately 1,700,000 hectares, with 80% situated in the country’s southern region. Puglia covers about 370,000 hectares, followed by Calabria and Sicily. Together, these three regions contribute to over 60% of Italy’s olive oil production [1]. However, this large production results in a proportional amount of organic by-products that need to be discharged [2]. The major by-products from the virgin olive oil chain are olive pomace and wastewater. Between these, of particular interest is olive pomace, representing a solid olive-processing by-product characterized by a complex of olive stones, skin, and pulp. Approximately 0.5–0.6 tons of pomace is produced for every ton of processed olives, with a moisture content of 50–65% based on the type of decanter used [3]. This olive pomace is mainly used to recover residual oil by solvent extraction. In particular, when this by-product is dried and subjected to solid–liquid extraction with hexane, exhausted olive pomace (EOP) is obtained [4]. In the same way as olive pomace, EOP consists of olive stones, pulp, and skin fragments, but it is characterized by an oil content lower than 2% and moisture of about 10% [5]. Thanks to the presence of stones, EOP has a high heating value (3755 kcal/kg), thus representing low-cost renewable fuel [5], while the generated ashes are widely employed to produce ceramic material [6]. However, both these employments generate emissions of hazardous gases and particles during combustion, resulting in environmental pollution [7], and do not allow the by-product to be used to its full potential. Hence, alternative strategies for the valorization of EOP have been proposed, such as the production of xylanases or the obtainment of ethanol, xylitol, and sugars from lignin or their polymeric sugars [6], and also using it as a source of phenolic compounds. Nowadays, the extraction of specialized molecules from plant by-products represents not only an important issue in nutraceutical fields, thanks to their antioxidant, anti-inflammatory, and chemopreventive properties, but also a strategy for reducing soil pollution. It is widely recognized that, if discarded, agro-industrial wastes may release phenolic compounds into the soil, with ecological consequences for plant growth and development, mainly affecting the germination stage [8]. Considering all these aspects, this investigation aims to compare different strategies of specialized molecule green extractive methods for EOP to confer additional value to this olive oil production by-product. Previous studies reported the recovery of phenolic compounds from EOP using maceration [6], decoction [9], ultrasound-assisted extraction (UAE) [6,10], and microwave-assisted extraction (MAE) [11]. However, there is no comparison between the cited extractive methods, designed to identify the most suitable one for the extraction of phenolic compounds from EOP. For this reason, this investigation aimed to compare the efficacy of different extractive methods such as maceration, UAE, MAE, and Accelerated Solvent Extraction (ASE), which, as far as is known, has never been applied to the extraction of active metabolites from EOP. 

When extracting bioactive compounds, it is important to consider not only the extraction method applied but also the type of solvent used, as it must have a minimal impact on the environment and human health. Among the solvents used, ethyl acetate, ethanol, and acetone were authorized by the European Food Safety Authority (EFSA) for functional food formulation [12]. Although these organic solvents have traditionally been touted as a safe and beneficial option, there is growing interest in finding more environmentally friendly alternatives to reduce the volatile organic compounds emission from these organic solvents, which have been known to contribute to global warming [13]. Previous investigations have identified water as the best solvent for recovering bioactive compounds from EOP [9] due to its non-selective nature. However, binary solvents (alcohol and water) have been demonstrated to be more effective in phenolic compound extraction than mono-solvent ones [14]. Specifically, ethanol/water mixtures appear to be the most appropriate extraction solvents, because of the distinct polarity of both solvents, the feasibility of combining them in any ratio, and their acceptability for human use [14]. For this reason, it was decided to compare the extractive efficacy of water alone and with the addition of a low percentage of ethanol (15%), since a previous investigation demonstrated that a solvent mixture with water as the prevalent solvent results in a low environmental impact [15]. All the obtained extracts were tested for their antioxidant activity using an in vitro cell-free assay, and those with the highest activity were also investigated for their antidiabetic potential via spectrophotometric assays and for their safety and antioxidant activity on human hepatoma cell lines (HepG2). Finally, the phytochemical profile of EOP’s most active extracts was determined.

Therefore, this investigation aims to find the best environmentally friendly extractive method for recovering active metabolites from olive by-products, to obtain extracts with the highest activity and thus confer a nutraceutical value to EOP in agreement with the concept of a green economy.

## 2. Results and Discussion

### 2.1. Extractive Yield 

The extractive solvent choice represents the first variable to be considered in any extraction method, since the yield and quantity of phenolic compounds recovered from a vegetal matrix are highly related to the solvent characteristics. The conventional solvents used are alcohols (methanol and ethanol), ethyl acetate, acetone, and diethyl ether, which can be mixed with water in different proportions. However, several disadvantages characterize the use of these solvents; in addition to a dangerous possible effect on human health, solvent residues may also persist in the final products. Hence, additional extract purification steps are demanded, with a consequent loss of time and an increase in the final total production costs [16]. It is also important to consider that organic solvents are not environmentally friendly; hence, in the last years, special attention has been directed toward using solvents that are not harmful to the environment or humans, such as water or a mixture of ethanol and water. However, to increase the efficiency of such solvents, it is necessary to optimize the extraction process using unconventional extraction techniques. Extractive methods can be distinguished into conventional ones like maceration, decoction, percolation, or infusion, and unconventional or advanced ones such as MAE, UAE, ASE, or supercritical fluid extraction [17]. Recently, the extraction of secondary metabolites from the EOP of *Olea europaea* L. has been optimized using MAE [11] and UAE [6]; however, as far as is known, there is a lack of an extraction methods comparison aimed to identify which best suits the extraction of secondary metabolites from EOP. Hence, this study compared MAC, as a conventional extractive technique, and MAE, UAE, and ASE, as unconventional extractive techniques, to verify the most efficient ones using water and 15% EtOH/water as solvent. Specifically, it obtained similar yields comprised of between 4.7 ± 0.31% and 6.8 ± 0.33% for hydroalcoholic extractions and 4.8 ± 0.39% and 6.1 ± 0.46% for water extractions (Table 1).

### 2.2. Total Phenolic Content (TPC) and Antioxidant Activity

As the first qualitative screening, the total phenolic content of EOP’s extracts was evaluated through the Folin–Ciocalteu assay, through which it is possible to evaluate not only the phenolic content of an extract but also its antioxidant activity. TPC is, indeed, an antioxidant test based on electron transfection, leading to the measurement of reductive antioxidant capacity. Data from the TPC evaluation are reported in Table 2. 

The TPC values obtained using UAE as an extraction technique were higher than those of the other investigations, which used the same method for obtaining EOP extracts. In the literature, a total phenolic content of 11.90 ± 0.30 mgGAE/100 g EOP was obtained using 47% ethanol/water [10] and 44.59 ± 1.46 mgGAE/g using 50% acetone/water [6]. Contrarily, comparisons with ASE cannot be made, since this extraction method was applied to EOP for the first time in the present study. As far as is known, a pressurized liquid extraction has been applied only on olive pomace [18] and not on exhausted olive pomace.

Antioxidant activity was also evaluated with two other spectrophotometric assays, the FRAP and DPPH assays, as, being based on oxidation–reduction reactions, they fitted with the TPC assay. The antioxidant values obtained using UAE as the extraction method and 15% ethanol/water as the extraction solvent (65.92 ± 1.62 and 121.17 ± 10.81 mgTE/g for DPPH and FRAP, respectively) are significantly superior to those obtained from UAE and 50% acetone (34.16 ± 0.33 and 59.27 ± 2.29 mgTE/g for DPPH and FRAP, respectively) [6]. From this observation, it is possible to speculate that a binary solvent with ethanol might represent a more suitable system than those containing acetone for EOP antioxidant molecule extraction. However, when water and UAE were applied, a higher antioxidant activity was obtained in the literature than in the present investigation. For instance, Vidal et al. [19] reported an antioxidant activity of 92.71 ± 0.39 mgTE/g and 147.41 ± 0.66 mgTE/g for DPPH and FRAP, respectively. However, it must be taken into account that the observed differences between studies could be due to the significant variability in the provenance of exhausted virgin pomaces, since they may be obtained from different oil production methods and degreasing procedures, thus affecting their phytochemical profile and the evaluated biological activity. On the other hand, the BCB test yielded divergent results, since, in this case, MAC gave the best ones with an antioxidant activity of 70.06 ± 5.26 and 80.89 ± 7.12% AA at 0.125 mg/mL for aqueous and hydroalcoholic extracts, respectively. This disparity can be attributed to the distinct principle of the assays used. In fact, while the FRAP and DPPH assays provide insight into the phenolic compounds’ reducing power and radical-scavenging activity, the BCB assay specifically assesses the possible inhibition of lipid peroxidation. Therefore, it is possible to suppose that extraction at atmospheric temperature and pressure, applicable with maceration, allows the extraction of active molecules involved in lipid peroxidation reduction that would otherwise not be extracted using the other methods. To the best of our knowledge, this is the first investigation evaluating the antioxidant activity of EOP extracts with the BCB assay. 

The Pearson correlation index was determined to evaluate a possible linear relationship between the antioxidant assays employed. As reported in Table 3, the highest correlation was obtained between TPC and FRAP (r = 0.86, 95%CI = 0.39–0.97; *p*-value = 0.006), TPC and DPPH (r = 0.77, 95%CI = 0.14–0.96; *p*-value = 0.025), and DPPH and FRAP (r = 0.88, 95%CI = 0.46–0.98; *p*-value = 0.004), confirming the complementarity that exists between the tests. In contrast, the BCB essay, due to its different nature, had no relation to any of the other essays, thus also corroborating the different results obtained.

**Figure 1:**
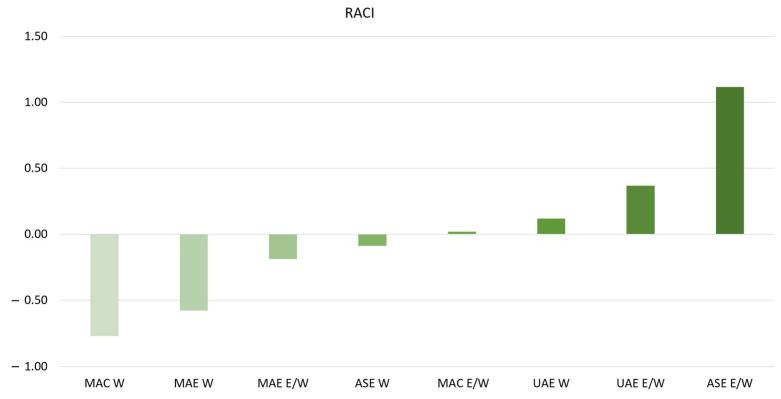
Relative Antioxidant Capacity Index (RACI) of aqueous (W) and hydroalcoholic (15%E/W) extracts obtained by different extraction techniques: maceration, MAC; ultrasound-assisted extraction, UAE; microwave-assisted extraction, MAE; Accelerated Solvent Extraction, ASE.

Hence, considering that different antioxidant assays may yield varying results based on the type of reagent and the underlying principles used, the RACI was determined to obtain a comprehensive overview of the extract’s activity [20]. RACI is a statistical index correlating the data obtained from several tests expressed with diverse measurement units (Figure 1). 

Applying the RACI, the hydroalcoholic extractions made using ASE and UAE techniques demonstrated the greatest values, highlighting the extracts’ strongest antioxidant activity. Hence, these two extracts were further investigated for their hypoglycemic activity, their antiradical activity through an in vitro cell-based assay, and their phytochemical profile. 

### 2.3. Hypoglycemic Activity

α-amylase and α-glucosidase enzyme inhibition is considered a treatment strategy for diseases such as obesity and diabetes, since they are involved in the digestion of complex carbohydrates. Therefore, the inhibitory effect of ASE and UAE hydroalcoholic extract was evaluated, demonstrating that both were able to inhibit the α-amylase enzyme, with an IC_50_ of 0.12 ± 0.01 mg/mL and 0.44 ± 0.02 mg/mL, respectively. However, a lack of activity was evidenced for α-glucosidase. Although the evaluated inhibitory activity against α-amylase is lower than that of the natural inhibitor acarbose, used as a positive control (IC_50_ of 0.02 ± 0.001 mg/mL), the obtained IC_50_ values of ASE and UAE hydroalcoholic extracts are lower than those evaluated for EOP hydroxytyrosol-rich fractions (IC_50_ of 3.0 ± 0.1 mg/mL) [21]. The higher activity of the extract evaluated in the present investigation suggests that the inhibition of α-amylase may be related not only to hydroxytyrosol but to the whole phytocomplex, which may include verbascoside, oleuroperin, or oleacein [21]. The data obtained are of the greatest interest, since it is known that, under certain circumstances, such as the excess activity of α-amylase and insulin resistance or deficiency, blood glucose levels can rise, with the consequent outbreak of hyperglycemic conditions [22]. Hence, this investigation confers to EOP a new nutraceutical value.

### 2.4. Effect of EOP Extracts on Cell Viability and Intracellular ROS

**Figure 2:**
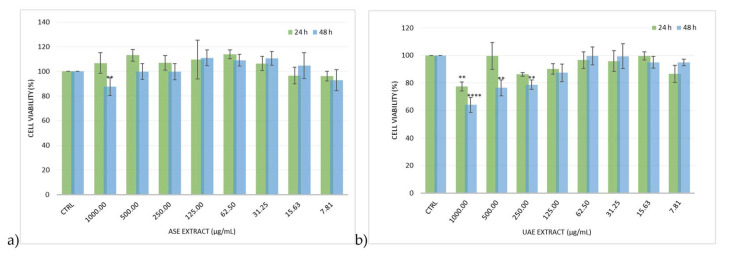
Cell viability, evaluated by MTT assay, of HepG2 cells treated for 24 and 48 h with different concentrations of olive pomace hydroalcoholic extract obtained by (**a**) Accelerated Solvent Extraction (ASE) and (**b**) ultrasound-assisted extraction (UAE). Data are expressed as the mean ± SD of three independent experiments (*n* = 3). **** *p* < 0.0001, ** *p* < 0.01 vs. control (CTRL).

The human hepatoma cell line (HepG2) is frequently used to evaluate potential plant extract cytotoxicity since, although HepG2 cells do not fully represent human hepatocytes, they retain a high mitochondrial activity and most of the human hepatocyte specialized characteristics. Therefore, this cell line was employed to evaluate the potential toxicity and antioxidant activity of UAE and ASE hydroalcoholic EOP extracts [23]. Cellular toxicity was investigated using the MTT assay, which revealed that ASE and UAE hydroalcoholic extracts did not exhibit toxicity. A decrease in cell viability was only observed at 1000 µg/mL, with less than a 10% reduction for the ASE hydroalcoholic extract and a less than a 40% reduction for the UAE hydroalcoholic extract (Figure 2a,b). However, it is essential to underline that this concentration is not reachable in human plasma, indicating that the extracts can be considered safe. 

**Figure 3:**
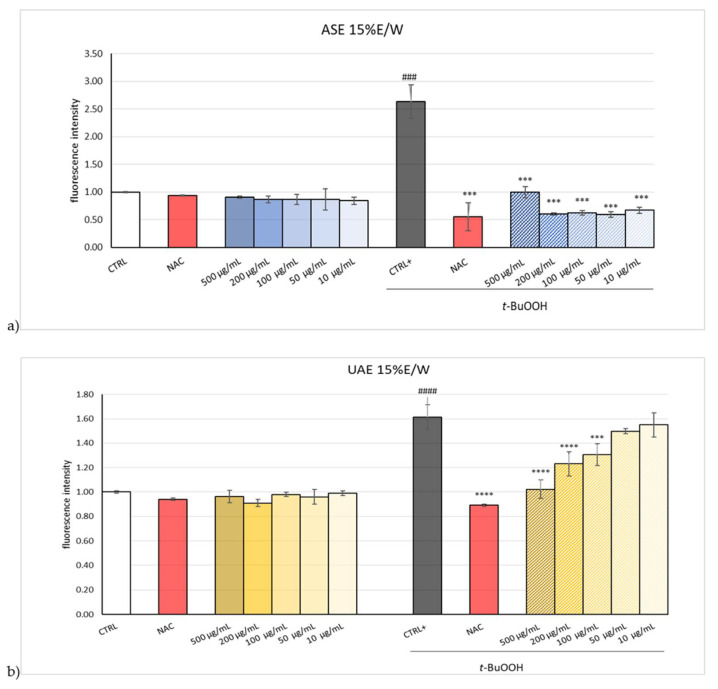
Intracellular ROS, evaluated by DCFH-DA fluorescent dye, in HepG2 cell line treated with different concentrations of olive pomace extracts, obtained by hydroalcoholic (15%E/W) extraction with (**a**) Accelerated Solvent Extraction (ASE) and (**b**) ultrasound-assisted extraction (UAE), for 24 h. *N*-acetylcysteine (NAC) was used as a positive control. Data are expressed as the mean ± SD of three independent experiments (*n* = 3). #### *p* < 0.001, ### *p* < 0.001 vs. control (CTRL), **** *p* < 0.0001, *** *p* < 0.001, vs. tert-Butyl hydroperoxide (*t*-BuOOH)-treated cells. Control treated with *t*-BuOOH (CTRL+).

The lack of cytotoxicity was also confirmed in a previous investigation, where EOP was tested in rat hepatoma FaO cells [24]. Based on this result, UAE and ASE hydroalcoholic EOP extracts were investigated for their antioxidant activity on HepG2 exposed to *terz*-butyl hydroperoxide (*t*-BuOOH), a known organic hydroperoxide. It was seen that EOP extracts reduced *t*-BuOOH oxidative stress induction in HepG2 cells, as depicted in Figure 3a,b. 

Specifically, it was seen that the HepG2 treatment with *t*-BuOOH resulted in a two-fold increase in fluorescence, indicative of an increase in intracellular oxidative stress. However, after cell treatment with ASE and UAE hydroalcoholic EOP extracts, the cells’ redox status returned to the basal level. Notably, the activity of the ASE hydroalcoholic extract is comparable to *N*-acetylcysteine (NAC), a recognized antioxidant, also at the lowest concentration (10 µg/mL), whereas the UAE extract could restore the redox status at 500 µg/mL and 200 µg/mL. The evaluated extracts’ beneficial effects on HepG2 cells could be attributed to tyrosol and hydroxytyrosol, known as cellular antioxidants capable of accumulating in the intracellular compartment to exert their activity [25].

### 2.5. Phytochemical Characterization 

ASE and UAE hydroalcoholic EOP extracts were evaluated for their chemical characterization. As determined by the standards’ UV spectra and retention times, seven phenolic molecules (Table 4) were detected at 280 nm in the two pomace extracts. A wavelength of 280 nm is commonly used to analyze phenols, since it allows the detection of a broad array of such molecules. The phenolic compounds identified in the extracts were gallic acid (1), 3-hydroxytyrosol (2), 3,4-dihydroxybenzoic acid (3), salidroside (4), catechin (5), tyrosol (6), and vanillic acid (7). 

**Figure 4:**
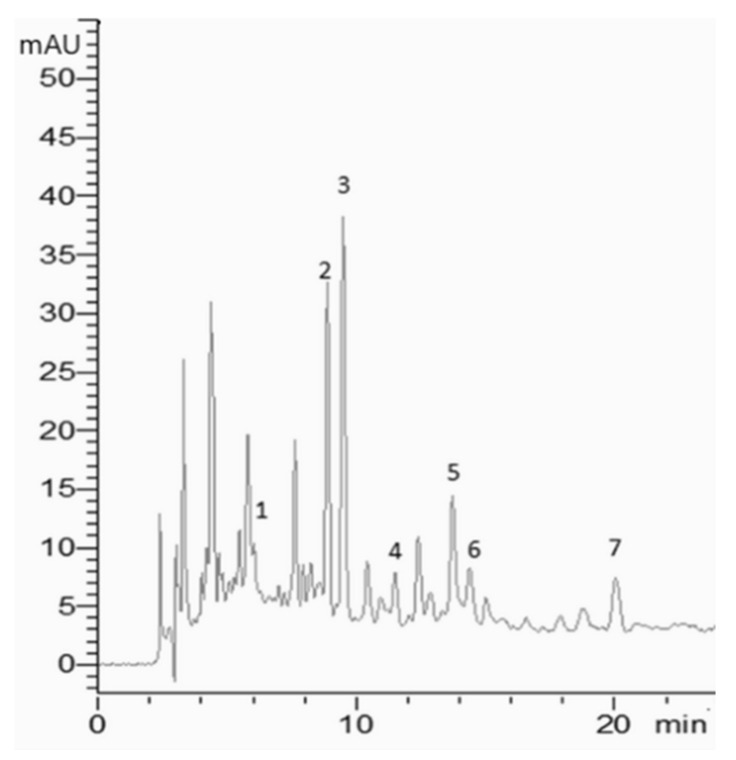
Exemplificative HPLC-DAD chromatogram of exhausted pomace extract obtained by hydroalcoholic Accelerated Solvent Extraction (ASE) recorded at 280 nm. Identified compounds **1**, gallic acid (tr = 5.98 min); **2**, 3-hydroxytyrosol (tr = 8.82 min); **3**, 3,4-dihydroxybenzoic acid (tr = 9.38 min); **4**, salidroside (tr = 11.42 min); **5**, catechin (tr = 13.65 min); **6**, tyrosol (tr = 14.27 min); **7**, vanillic acid (tr = 19.87 min).

Notably, if ASE and UAE hydroalcoholic extracts showed the same phytochemical profile from a qualitative viewpoint, the ASE extract was demonstrated to be the richest extract from a quantitative point of view. Considering this aspect, the ASE hydroalcoholic extract chromatogram is reported as an example (Figure 4).

Hydroxytyrosol was one of the most abundant active molecules in the ASE and UEA hydroalcoholic EOP extracts, confirming data from Gómez-Cruz et al. (2020) [9]. However, compared to this last investigation, our current one found the greater amount. This discrepancy may be attributed to the different solvents used, since Gómez-Cruz et al. employed water as solvent extraction, obtaining an amount of hydroxytyrosol ranging between 5.73 and 9.12 mg/g EOP [9]. In contrast, in the present investigation using 15% EtOH, 13.57 ± 0.51 and 11.41 ± 0.42 mg/g of hydroxytyrosol were found in the ASE and UAE EOP extracts, respectively. Furthermore, this amount was near those found by Vidal et al. [19], which in concentrated phenolic EOP extracts quantified 16.69 ± 0.00 mg/g of hydroxytyrosol. In the same way, regarding tyrosol, a similar concentration was found in the present investigation (3.01 ± 0.19 and 4.03 ± 0.93 mg/g for UEA and ASE hydroalcoholic EOP extracts, respectively) and in concentrated phenolic EOP extracts (2.08 ± 0.01 mg/g) [19].

**Figure 5:**
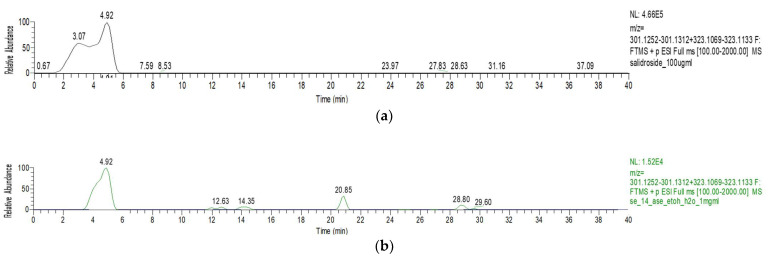
HR-ESI-MS-HPLC analysis of olive pomace extracts: (**a**) Extracted-ion chromatograms at *m*/*z* 301.1282 + *m*/*z* 323.1101 of salidroside from standard sample at 100 µg/mL; (**b**) extracted-ion chromatograms at *m*/*z* 301.1282 + *m*/*z* 323.1101 of salidroside from hydroalcoholic Accelerated Solvent Extraction (ASE 15%E/W) sample at 1 mg/mL.

Noteworthy is the presence of salidroside, a tyrosol glucoside normally found only in olives [26], which was found for the first time in EOP in this study at the concentration of 6.70 ± 0.29 and 7.94 ± 0.77 for the UEA and ASE hydroalcoholic EOP extracts. Thus, an LC-HR-MS analysis of the two EOP extracts was performed to confirm the presence of this active compound, to demonstrate that it is present in either UEA or ASE extracts with a retention time of 4.92 min. Figure 5 displays the extracted-ion chromatograms of the ASE hydroalcoholic EOP extract. 

Salidroside has long been used as an adaptogen in traditional medicine and is highly valued for its diverse pharmacological properties. It is known for its stimulant, tonic, cardioprotective, anticancer, neuroprotective, immune-stimulant, and anti-inflammatory properties [27]. However, the availability of salidroside is complicated, since it is primarily extracted from *Rhodiola* species, where the content is generally less than 1% [27]. Hence, using by-products from olives to extract this valuable compound represents an excellent opportunity, reinforcing the need to valorize it. 

## 3. Materials and Methods

### 3.1. Chemicals and Reagents

Ethanol was acquired from Carlo Erba (Milan, Italy), while analytical grade methanol was purchased from Merck (Darmstadt, Germany and Mollet del Vallés, Spain). Sodium carbonate, 2,2-diphenyl-1-picrylhydrazyl (DPPH), Folin–Ciocalteu reagent, potassium phosphate monobasic, iron (III) chloride (FeCl_3_·6H_2_O), butylated hydroxytoluene (BHT, 2,6-bis (1,1-dimethylethyl)-4-methylphenol), Tween 20, linoleic acid, *β*-carotene, ascorbic acid, 6-hydroxy-2,5,7,8-tetramethylchroman-2-carboxylic acid (Trolox), gallic acid, acarbose, starch, *α*-amylase enzyme from porcine pancreas, potassium iodide (KI), iodine (I_2_), 4-p-nitrophenyl-*α*-D-glucopyranoside, glutamine, *α*-glucosidase enzyme from Saccharomyces cerevisiae, fetal bovine serum (FBS), potassium phosphate monobasic, sodium carbonate, 2-deoxy-2-[(7-nitro-2,1,3-benzoxadiazol-4-yl)amino]-d-glucose (2-NBDG), Dulbecco’s modified Eagle’s medium (DMEM), streptomycin, 3-(4,5-dimethylthiazol-2-yl)-2,5-diphenyltetrazolium bromide (MTT), and penicillin were acquired from Sigma (St. Louis, MO, USA, and Steinheim, Germany). Analytical standards were purchased from Merck (Wicklow, Ireland) and Sigma-Aldrich (Milan, Italy). 

### 3.2. Equipment 

UV–Vis spectrophotometer (SPECTROstarNano BMG Labtech, Ortenberg, Germany). Shimadzu HPLC system (LC-20AB, Prominence Diode Array Detector, Shimadzu Corporation, Kyoto, Japan) coupled with a Diode Array Detector and equipped with a binary pump. Thermo LTQ Orbitrap XL high-resolution ESI mass spectrometer coupled to Thermo U3000 HPLC system. Flow cytometer (FACS CANTO II, Becton Dickinson, Sunnyvale, CA, USA).

### 3.3. Sample Collection and Extraction

The collection of exhausted olive pomace (EOP) from *Olea europaea* L. was carried out in November 2018 from an olive mill located in Venosa, Potenza, Italy, considering the seasonality of olive oil production. Samples were frozen at −20 °C to preserve their integrity.

The extraction process was optimized by comparing four different extractive methods and two solvent ratios. Each extraction was performed three times, with fresh solvent, to recover the active compounds from the matrix completely. The extracts obtained from each replicate were mixed, filtered with a vacuum pump equipped with filters of 40 µm, and subsequently subjected to drying through a rotary evaporator. Finally, the samples were frozen and subjected to freeze-drying. The extraction yield was calculated and expressed as a percentage of dried extract (de).

#### 3.3.1. Maceration (MAC)

Maceration is a conventional method where the vegetable material is soaked in a solvent, allowing the soluble components to dissolve and be extracted from the cellular structure. In this study, in amber bottles, 15 g of EOP was subjected to maceration using 80 mL of solvent (either 100% water or a mixture of 15% ethanol in water). The maceration process was carried out under agitation, in the absence of light, and at room temperature for 2 h.

#### 3.3.2. Ultrasound-Assisted Extraction (UAE)

Ultrasound-assisted extraction is a technique that relies on the phenomenon of cavitation, which causes the disruption of the cell walls of plants and enhances the metabolites’ transfection into the solvent used for the extraction [28]. In line with the procedures outlined by Goldsmith et al. (2018) [28], 15 g of EOP was subjected to sonication in a dark glass bottle by adding 80 mL of solvent (either a mixture of 15% ethanol in water or 100% water). The beaker was lidded with a glass cap, and the sonication process was performed using a Bransonic M1800-E sonicator operating at 40 kHz frequency and 70 W power output. The duration of the sonication was 2 h. The temperature, monitored at 30 min intervals, varied between 25 and 40 °C during the extraction process.

#### 3.3.3. Microwave-Assisted Extraction (MAE)

Microwave extraction is a method that utilizes electromagnetic radiation to heat solvents, to extract analytes from the vegetable matrix. Following a previously validated method described by Russ, et al. [29], in a dark glass bottle, 80 mL of solvent (either 100% water or a mixture of 15% ethanol in water) was combined with 15 g of EOP. The beaker was then covered with parafilm to ensure a sealed environment. The process of extraction was carried out using a microwave oven with a power output of 100 W. The extraction procedure consisted of an initial microwave treatment for 5 min, followed by a subsequent 25 min period at room temperature. This process was repeated during a complete two-hour extraction time. 

#### 3.3.4. Accelerated Solvent Extraction (ASE)

Accelerated Solvent Extraction (ASE) is a method that enables shorter extractive times by utilizing high pressures and temperatures and small volumes of solvent. In this study, 12 g of EOP was subjected to extraction using an accelerated solvent extractor (ASE 150, Dionex). The solvents used were either 100% water or a mixture of 15% ethanol in water. The extraction conditions involved a steel extraction cell of 22 mL, a temperature of 80 °C, and a pressure ranging from 103.42 to 110.31 bar. The extraction process consisted of three static extraction cycles, each lasting 5 min.

### 3.4. Polyphenol Content and Antioxidant Activity

#### 3.4.1. Total Polyphenolic Content (TPC)

The total phenolic content (TPC) was assessed by the Folin–Ciocalteu method, using the assay detailed by Uddin et al. (2022) [23]. Based on a standard curve, data were expressed as milligrams of gallic acid equivalents (GAEs) per gram of dried extract (mg GAE/g).

#### 3.4.2. DPPH Assay

The 2,2-diphenyl-1-picrylhydrazyl (DPPH) assay is based on DPPH solution reduction by an antioxidant that donates hydrogen atoms, resulting in the formation of the non-radical diphenylpicrylhydrazine (DPPH-H). This reduction is dose-dependent and is accompanied by a colorimetric change from purple to yellow. The extent of the color change can be measured spectrophotometrically at a wavelength of 515 nm [30]. The test was made following the protocol of Milella et al. (2014) [31]. Based on a standard curve, the results were expressed as milligrams of Trolox equivalent (TE) per gram of dried extract (mg TE/g).

#### 3.4.3. FRAP Assay

The FRAP assay is a method assessing a sample’s antioxidant capacity by measuring its ability to reduce the ferric tripyridyltriazine (Fe^3+^-TPTZ) complex to the ferrous form (Fe^2+^-TPTZ). The Fe^2+^-TPTZ complex forms an intense blue color, which can be measured at an absorbance maximum of 593 nm. The increase in absorbance at 593 nm is proportional to the total reducing power of the electron-donating antioxidants present in the sample. The essay was performed based on the protocol of Libutti et al. (2023) [32]. Based on a standard curve, the results were expressed as milligrams of Trolox equivalent (TE) per gram of dried extract (mgTE/g).

#### 3.4.4. BCB Assay

The BCB assay measures the capacity of antioxidants to inhibit the oxidation of *β*-carotene in the presence of free radicals. The principle relies on the fact that *β*-carotene, when exposed to oxidative stress (usually in the form of hydrogen peroxide or free radicals), degrades and loses its color. Antioxidants in the sample can prevent or slow down this degradation process. The lipid peroxidation inhibition was assessed using the *β*-carotene bleaching method (BCB), considering the protocol described by Condelli et al. (2015) [33]. 

#### 3.4.5. Relative Antioxidant Capacity Index (RACI)

The Relative Antioxidant Capacity Index (RACI) is a dimensionless index used in statistics to compare and integrate data from distinct in vitro antioxidant methods. It addresses the challenge of combining data from various assays with different units and scales, allowing for a more comprehensive antioxidant capacity comparison. The RACI was calculated considering the procedure of Libutti et al. (2023) [32].

### 3.5. Inhibition of α-Amylase and α-Glucosidase

The extracts’ ability to inhibit enzymes involved in carbohydrate digestion, such as α-amylase and *α*-glucosidase, was evaluated, taking into consideration the protocol of Faraone et al. (2021) [34]. 

### 3.6. In Vitro Cell-Based Assays 

#### 3.6.1. Cell Culture Maintenance and Treatments

Human hepatoblastoma cell line, HepG2 cells, were cultured in Dulbecco’s modified Eagle’s medium (DMEM) with the addition of 10% fetal bovine serum (FBS), 25 mM glucose, 2 mM L-glutamine, 100 U/mL penicillin, and 100 µg/mL streptomycin. Cells were stored at 37 °C in a humidified atmosphere with 5% CO_2_.

Immortalized human hepatocyte (IHH) cells were cultured in DMEM F-12 with the addition of 1% penicillin (100 IU/mL), 10% FBS, 1 μM dexamethasone, 10–12 M insulin, and 100 μg/mL streptomycin. IHH cells were stored at 37 °C with 5% CO_2_ in a moist atmosphere.

Extracts were prepared to the desired concentration with DMEM prior to use in the experiments.

#### 3.6.2. Cell Viability Assay and Intracellular Reactive Oxygen Species (ROS)

An MTT (3-(4,5-dimethylthiazol-2-yl)-2,5-diphenyltetrazolium bromide) assay was used to assess cell viability. HepG2 cell lines were seeded to a density of 1.5 × 10^4^ in a 96-well culture plate and treated with distinct extract concentrations (10 µg/mL to 500 µg/mL) for 24 and 48 h. The MTT assay measures the yellow MTT reduction by mitochondrial reductase activity to a purple formazan product. The absorbance of the formazan product was quantified at 570 nm using a microplate reader (SPECTROstarNano BMG Labtech, Ortenberg, Germany), with a background subtraction at 630 nm. The OD_570_-OD_630_ value is directly proportional to the viable cell number. The treated cells’ viability percentage was determined as follows:

(1) $$\%viability\;of\;cells=\frac{Average\;optical\;density\;of\;treated\;cells}{Average\;optical\;density\;of\;control\;cells}\times 100$$

The intracellular ROS were determined using a fluorescent probe (2′-7′-dichlorofluorescein) and flow cytometer system (FACS CANTO II, Becton Dickinson, Sunnyvale, CA, USA) as reported by Sinisgalli et al. (2020) [35].

### 3.7. High-Performance Liquid Chromatography–Diode Array Detector (HPLC-DAD) 

The phytochemical profile of EOP was performed using a Shimadzu HPLC system (LC-20AB, Prominence Diode Array Detector, Shimadzu Corporation, Kyoto, Japan) coupled with a Diode Array Detector and equipped with a binary pump. Extracts were dissolved in deionized water and filtered using polytetrafluoroethylene syringe filters of 0.2 µm. Chromatographic separation was performed using a Nucleodur C18 reversed-phase column (250 mm × 4.6 mm, 5 µm) from Macherey-Nagel (67722 Hoerd, France), using a gradient elution program consisting of a gradient between water (Phase A) and methanol (Phase B), both with 5% formic acid at 1 mL/min as the flow rate. The elution was conducted as follows: a linear increase in 3 min from 5% to 15% B, in 22 min from 15% to 25% B, in 5 min from 25% to 55% B, and in 5 min from 55% to 75% B, followed by column washing and reconditioning. The injection volume was 20 μL. Thirty standards prepared in methanol were injected using the same chromatographic conditions employed for the samples. The phytochemical profile of the olive pomace extracts was defined based on the standards’ retention times and UV–visible spectra. The data analysis was performed using LabSolutions software version 5.51 (Shimadzu Corporation, Kyoto, Japan).

### 3.8. LC-MS Analysis

The LC-MS analysis was performed through a Thermo LTQ Orbitrap XL high-resolution ESI mass spectrometer coupled to Thermo U3000 HPLC system. Chromatographic separation was accomplished with a 5 μm Kinetex C18 column (50 mm × 2.10 mm). The elution was performed at a 200 μL/min flow rate using a gradient elution with H_2_O acidified with 0.1% HCOOH (Phase A) and MeOH (Phase B) as mobile phases. The elution program consisted of an initial 3 min isocratic elution with 10% MeOH, followed by a linear increase to 100% MeOH in 30 min and maintenance for 7 min in 100% MeOH. The acquisition of mass spectra was made in positive ion detection mode. The MS parameters included a 4.8 kV spray voltage, a 32 units N_2_ (approximately 150 mL/min) sheath gas rate, 285 °C as the capillary temperature, and a 15 units N_2_ (approximately 50 mL/min) auxiliary gas rate. The results achieved from the analysis were analyzed using Thermo Xcalibur software (2.2 SP1 build 48).

### 3.9. Statistical Analysis

The experiments were performed in triplicate, and the results were reported as the mean ± standard deviation (SD). The statistical analysis was evaluated with Minitab 19 (©2024 Minitab, LLC, State College, PA, USA), using a one-way analysis of variance (ANOVA) to determine any significant differences between the samples, with a confidence level of 95%. Tukey’s test was employed to match the means and identify any significant differences, with a significance level set at *p* < 0.05. Furthermore, a correlation analysis was performed using Pearson’s correlation coefficient (r) to examine the relationships between variables.

## 4. Conclusions

Exhausted olive pomace represents the principal residue obtained from olive pomace, which can be used not only for bioenergy production but also as a source of active compounds with antioxidant and hypoglycemic activity. This investigation aims to compare different strategies for extracting specialized molecules from EOP to enhance the value of this by-product of olive oil production. Of all the extraction techniques tested, hydroalcoholic ASE proved to be the best one, providing extracts with the highest antioxidant and hypoglycemic activity, which were the richest in specialized molecules such as 3-hydroxytyrosol, tyrosol, and salidroside, identified for the first time in EOP extracts. Therefore, ASE performed with 15% ethanol as an extraction solvent may represent a suitable extractive technique for conferring a new nutraceutical valorization to EOP. The results of this study confirm that EOP has significant potential as an economical source of bioactive compounds and propose a new extraction method applicable at the industrial level. Further studies will be conducted to isolate EOP specialized molecules and evaluate their biological activity. 

## Figures and Tables

**Table 1:** Extractive yields expressed as percentage of dried extract (%de) as median ± standard deviation.

	50% E/W	W
UAE	6.80 ± 0.34 ^a^	6.16 ± 0.46 ^a^
MAE	5.95 ± 0.43 ^a,b^	5.39 ± 0.36 ^a,b^
ASE	5.12 ± 0.28 ^b,c^	4.85 ± 0.39 ^b,c^
MAC	4.74 ± 0.31 ^c^	3.90 ± 0.30 ^c^

Water, W; ethanol, E; maceration, MAC; ultrasound-assisted extraction, UAE; microwave-assisted extraction, MAE; Accelerated Solvent Extraction, ASE. Statistically significant differences (*p* < 0.05) are highlighted with different superscript letters (^a^, ^b^, ^c^).

**Table 2:** Results of total phenolic content and antioxidant activity of olive pomace extracts obtained by different extraction methods.

Samples	TPC mgGAE/g	DPPH mgTE/g	FRAP mgTE/g	BCB %AA at 0.125 mg/mL
MAC W	30.30 ± 2.06 ^e^	41.74 ± 3.48 ^d^	86.51 ± 8.68 ^d^	70.06 ± 5.26 ^a,b^
MAC 15%E/W	34.50 ± 1.93 ^d,e^	52.38 ± 5.70 ^c,d^	107.13 ± 10.84 ^b,c,d^	80.89 ± 7.12 ^a^
UAE W	52.66 ± 3.11 ^a,b^	63.70 ± 6.03 ^b,c^	112.16 ± 1.20 ^a,b,c^	39.61 ± 16.40 ^c,d^
UAE 15%E/W	59.25 ± 4.71 ^a^	65.92 ± 1.62 ^b^	121.17 ± 10.81 ^a,b^	32.51 ± 1.17 ^d^
MAE W	35.56 ± 1.51 ^d,e^	51.33 ± 2.98 ^c,d^	91.99 ± 4.44 ^c,d^	57.58 ± 10.29 ^b,c^
MAE 15%E/W	45.86 ± 3.83 ^b,c^	62.65 ± 4.20 ^b,c^	109.38 ± 8.12 ^a,b,c^	34.14 ± 4.14 ^d^
ASE W	40.65 ± 3.72 ^c,d^	53.69 ± 4.44 ^b,c,d^	110.42 ± 3.06 ^a,b,c^	58.11 ± 6.76 ^a,b,c^
ASE 15%E/W	54.32 ± 5.89 ^a,b^	94.67 ± 5.28 ^a^	129.95 ± 10.31 ^a^	50.22 ± 4.09 ^b,c,d^

Water, W; ethanol, E; maceration, MAC; ultrasound-assisted extraction, UAE; microwave-assisted extraction, MAE; Accelerated Solvent Extraction, ASE; total phenolic content, TPC; 2,2-diphenyl-1-picrylhydrazyl, DPPH; ferric reducing antioxidant power, FRAP; *β*-carotene bleaching assay, BCB. Each experiment was replicated three times (*n* = 3), and results are expressed as mean ± standard deviation. Statistically significant differences (*p* < 0.05) are highlighted with different superscript letters (^a^, ^b^, ^c^, ^d^, ^e^).

**Table 3:** Pearson correlation between TPC (total phenolic content), DPPH (2,2-diphenyl-1-picrylhydrazyl), FRAP (ferric reducing antioxidant power), and BCB (*β*-carotene bleaching assay).

	TPC	DPPH	FRAP	BCB
TPC		0.77	0.86	−0.82
DPPH	0.77		0.88	−0.48
FRAP	0.86	0.88		−0.50
BCB	−0.82	−0.48	−0.50	

**Table 4:** Quantification of compounds in ASE and UAE exhausted olive pomace extracts at 280 nm.

	Retention Time (min)	Compound	Mean ± Standard Deviation (mg/g)	
			UAE 15% E/W	ASE 15% E/W
1	6.01	Gallic acid	0.40 ± 0.18	0.41 ± 0.08
2	8.86	3-Hydroxytyrosol	11.41 ± 0.42	13.57 ± 0.51
3	9.38	3,4-Dihydroxybenzoic acid	14.54 ± 0.28	14.31 ± 1.07
4	11.49	Salidroside	6.70 ± 0.29	7.94 ± 0.77
5	13.74	Catechin	5.18 ± 0.01	6.46 ± 0.20
6	14.37	Tyrosol	3.01 ± 0.19	4.03 ± 0.93
7	20.03	Vanillic acid	0.38 ± 0.07	0.72 ± 0.01
Total amount			41.62 ± 1.44	47.44 ± 3.57

Ultrasound-assisted extraction, UAE; Accelerated Solvent Extraction, ASE.

## Data Availability

Data are available upon request.
